# ‘Things often get worse before they get better’: participants’ responses to the cessation of recruitment and trial treatment in a trial of a psychological therapy for people with personality disorder following a safety alert. (A missed opportunity for collaboration?)

**DOI:** 10.1186/1745-6215-14-S1-P94

**Published:** 2013-11-29

**Authors:** Florence Day, Mary McMurran, Lelia Duley

**Affiliations:** 1 Nottingham Clinical Trials Unit, University of Nottingham, Nottingham, UK; 2 Institute of Mental Health, University of Nottingham, Nottingham, UK

## 

The study team were advised by the independent oversight committees to stop trial recruitment and the delivery of trial therapy due to a difference in the number of reported hospitalisations between the treatment and control group.

Because of concerns over potential worry or distress for participants resulting from the trial changes, implementation was carefully monitored throughout. Discussions with participants were documented and feedback recorded whenever this was given. Feedback has been reviewed to identify learning points and enable reflection on the processes used in the trial and during implementation of the trial changes.

Specific feedback was received and documented from 37 participants from all trial stages. A number of recurring themes were identified. Among the most frequently reported were the view that an initial increase in distress was expected when engaging in psychological therapies, concern over lack of alternative treatment options, alternative interpretations of the findings and insights into both the processes by which therapy was delivered in the trial and the way in which the trial changes were implemented.

Feedback from participants provided useful insights and there are lessons to be learned for researchers and clinicians. Involving service users in a collaborative dialogue regarding the safety issues identified and the options for the trial may have been fruitful. There is scope for alternative understandings of safety issues in trials of psychological interventions in mental health and discussions about what constitutes safety in this context are warranted. Insights from service users should inform conceptualisations of safety in future psychotherapy trials.

